# Assessing the reproducibility and validity of a food frequency questionnaire for pregnant women from the Chinese Miao ethnic group

**DOI:** 10.3389/fnut.2024.1322225

**Published:** 2024-05-07

**Authors:** Xiaorong Ni, Tian Qiao, Rong Wang, Fang Wang, Yi Liang, Shaofeng Wei

**Affiliations:** ^1^The Key Laboratory of Environmental Pollution Monitoring and Disease Control, Ministry of Education, School of Public Health, Guizhou Medical University, Guiyang, China; ^2^Department of Clinical Nutrition, Affiliated Hospital of Guizhou Medical University, Guiyang, China

**Keywords:** food frequency questionnaire, reproducibility, validity, pregnant women, Chinese Miao ethnicity

## Abstract

**Background:**

Currently, no food frequency questionnaire is available to be administered exclusively to ethnic minorities in China. This study aimed to evaluate the reproducibility and validity of a culturally tailored semi-quantitative food frequency questionnaire (FFQ) designed for pregnant women belonging to the Miao ethnic group in China.

**Methods:**

A total of 74 questions in the FFQ were administered to collect dietary information from Miao women in China during their pregnancy. This study included 153 and 127 pregnant women, respectively, for testing the validity and reproducibility of the results. Baseline FFQ data (FFQ1) were collected initially, followed by the administration of a repeated FFQ 4–6 weeks later (FFQ2). Two 24-h recalls (24HR) were used as references to compare food groups and nutrient intake. Pearson/Spearman's coefficients were used to measure the validity and reproducibility of the FFQ. Quartile cross-classification, weighted kappa coefficients, and Bland–Altman plots were employed to assess the agreement.

**Results:**

Most food groups and nutrient intake estimated by the FFQ were higher than those estimated by the 24HR. Food groups and nutrients' correlations for FFQ vs. 24HR after being energy-adjusted and de-attenuated, respectively, were 0.10 (vegetables) to 0.45 (grains/tubers) and 0.15 (iron) to 0.52 (riboflavin). Comparatively, correlation coefficients for FFQ1 vs. FFQ2 ranged from 0.41 (fruit) to 0.71 (vegetables) and from 0.45 (energy) to 0.64 (calcium). The percentage of pregnant women classified in the same or adjacent quartiles ranged from 64.08% (vegetables) to 95.29% (sour soup) and from 68.88% (vitamin E) to 78.81% (energy). Weighted kappa coefficients exceeded 0.2 for food groups and most nutrients, and Bland–Altman plots demonstrated acceptable agreement between the two tools.

**Conclusions:**

This study provides novel information on the validation of FFQ. It demonstrates that the FFQ exhibits ideal reproducibility and acceptable validity in estimating and ranking the intake of food groups and most nutrients among pregnant women belonging to the Chinese Miao ethnic group.

## 1 Introduction

Nutrition during pregnancy is vital and significantly impacts maternal and fetus health. An imbalanced diet during pregnancy can increase the risk of gestational diabetes mellitus (GDM), hypertension, anemia, preeclampsia, premature birth, and low birth weight (1–5). The assessment of food intake during pregnancy can provide crucial insights into dietary adequacy. Food records (FR) and 24-h recalls (24HR) are effective methods for accurately documenting food consumption details. However, they are not frequently utilized in epidemiological studies due to the high cost involved and unrepresentativeness of short-term intake (6, 7). Food frequency questionnaire (FFQ) is commonly used to investigate associations between dietary exposure and diseases because of its cost-effectiveness, ability to assess long-term food preferences, and capacity to rank individuals based on their dietary intake (8, 9). However, FFQ should be validated, as it is sensitive to cultural, regional, ethnic, and dietary customs. An inappropriate FFQ can lead to erroneous estimates of food intake.

Zhang et al. (10) and Yuan et al. (11) evaluated the reproducibility and validity of FFQ in Chinese pregnant women, but their studies were conducted in northern, central, and western China (10–14). Specific dietary differences exist across regions and ethnic groups in China. To date, no validation study of the FFQ has been administered among pregnant women of Miao ethnicity in southwest China. The Miao ethnic group is the fifth largest minority group in China, with a population of 11.07 million (15), and exhibits distinctive dietary preferences (16). Their diet centers on polished rice as the staple, featuring common dishes like sour soup, pickled foods, and a range of stews. The Maternal and Child Cohort in China's Miao Ethnic Group (MCCMC), an effective assessment tool with a major objective of investigating the associations between dietary intake and adverse pregnancy outcomes, is particularly critical for this cohort. Therefore, we developed a new FFQ to estimate long-term dietary habits in this population and assessed its reproducibility and validity.

## 2 Materials and methods

### 2.1 Participants and study design

Participants' inclusion criteria include (a) singleton pregnant women from the Miao ethnic group; (b) those in the age of ≥18 years; (c) those who were proposed routine antenatal check-ups at the cohort centers; and (d) those residing within Miao tribes for more than 1 year. The exclusion criteria were pregnant Miao women diagnosed with malignant or chronic diseases before pregnancy, requiring strict dietary control or medical treatment. Pregnant women at different trimesters of pregnancy were invited to participate in our study. To determine the sample size for measuring the correlation between FFQ and 24HR, the following formula was used:


$$\begin{array}{l} n={\left(Z_{\text{α}}+Z_{\text{β}}\right)}^{2}\sigma^{2}/d^{2} \end{array}$$

**Figure 1:**
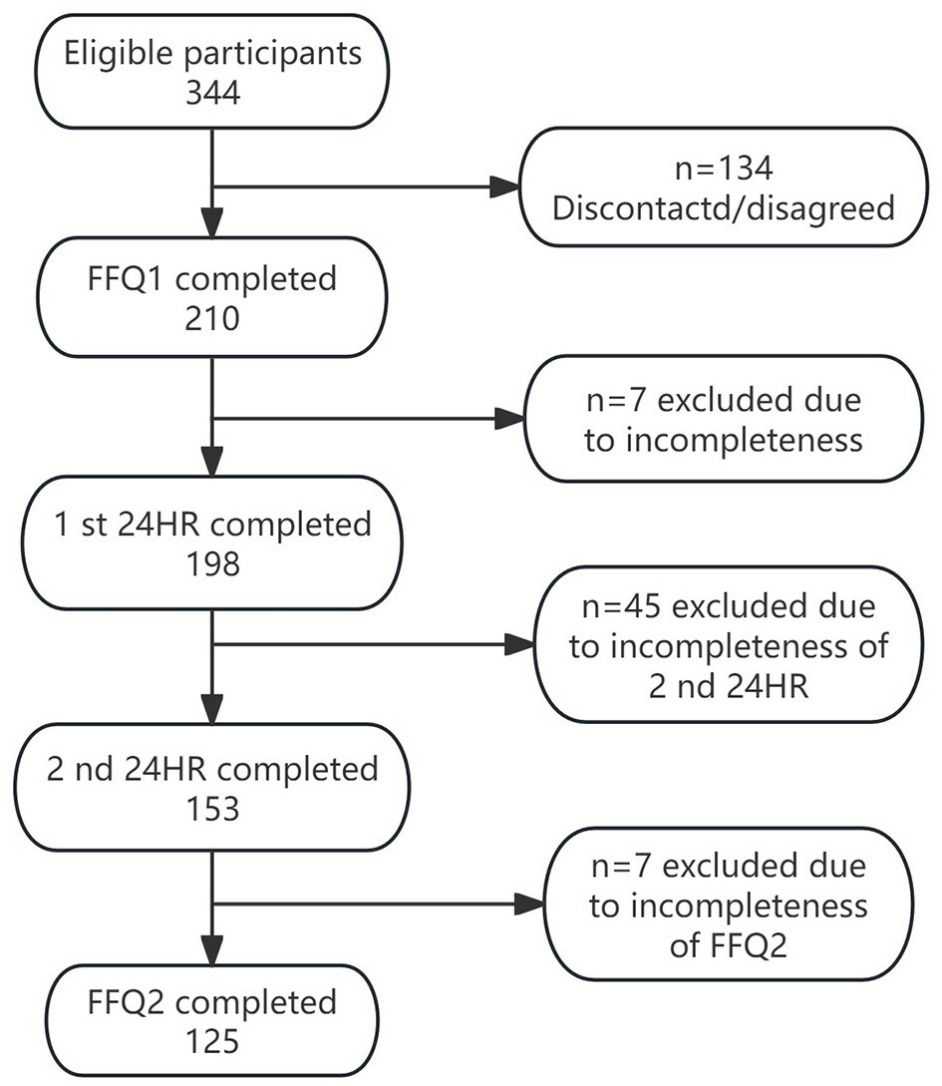
Flow diagram of sample selection. FFQ1, the first food frequency questionnaire; FFQ2, the second food frequency questionnaire (FFQ2); 24HR, 24-hour recalls.

With a significance level of 0.05 and a statistical power of 0.8, a minimum sample size of 110 was determined. We recruited pregnant women belonging to the Miao ethnic group using a convenience sampling method from July to September 2022 at two hospitals in Qiandongnan Miao and Dong Autonomous Prefecture, Guizhou Province, China. Initially, 344 pregnant women were invited. Of these, 125 and 153 pregnant women provided complete for two FFQs and two 24HR data, respectively (Figure 1).

**Figure 2:**
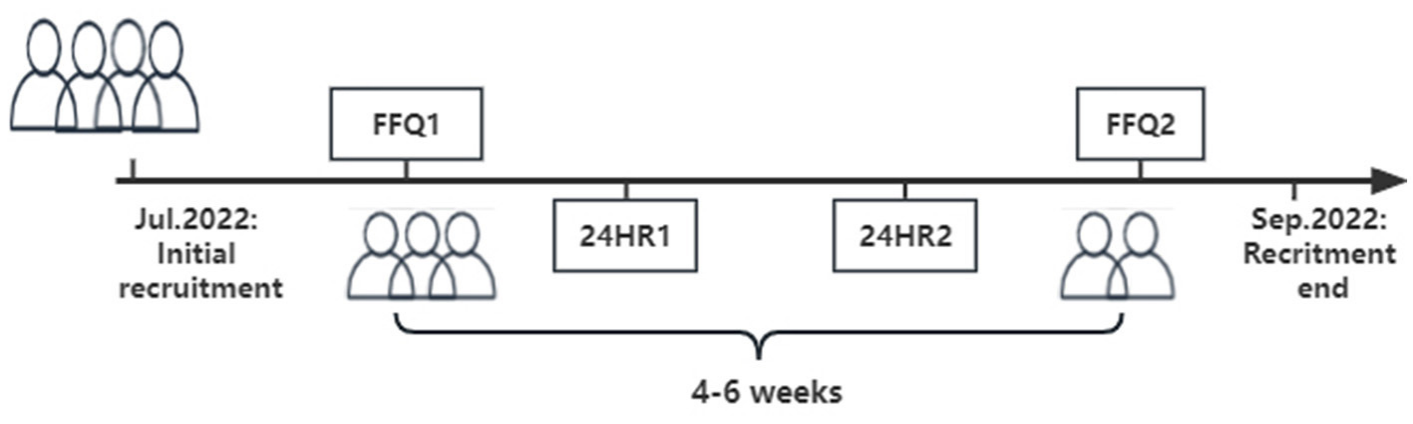
Design of reproducibility and validity in the present study. FFQ1, the first food frequency questionnaire; FFQ2, the second food frequency questionnaire (FFQ2); 24HR, 24-hour recalls.

For the reproducibility study, we collected data from the first FFQ (FFQ1) administered to pregnant women during their routine antenatal check-ups at the cohort centers. Subsequently, the second FFQ (FFQ2) was administered after 4–6 weeks. This time frame was deemed appropriate to minimize recall bias and consider the influence of seasonal variations on dietary differences (17). In the validity study, two non-consecutive 24HR (including 1 weekday and 1 weekend) were used as the reference method, and these recalls were conducted between FFQ1 and FFQ2 (Figure 2).

Data for the FFQ and 24HR were gathered via face-to-face interviews conducted by trained investigators, given the participants' limited literacy skills. Other demographic information was obtained verbally, except for height and weight, which were measured on-site by the obstetrician.

### 2.2 Ethics approval and informed consent

This study was approved by the Ethics Committee of the Affiliated Hospital of Guizhou Medical University, Approval no. 2021 (065–01). Written informed consent was obtained from the participants before the investigation.

### 2.3 FFQ and 24HR

Before formally developing the FFQ, our research team interviewed two nutritionists in Miao settlements. We gathered information about the common and distinctive food items consumed by the Miao population (food list), their frequency of daily meals, and meal times. Combining the collected food list with the Chinese food composition table (18), a new FFQ was developed eventually. It consisted of 74 food items categorized into six food groups according to the Chinese Resident Dietary Guidelines (19): grains/tubers, vegetables, fruits, and meat/poultry/fish/eggs/dairy. Additionally, sour soup, a hallmark of Miao dietary customs, encompassing both red and white varieties, was incorporated.

An open-ended format was selected to describe frequency. Participants were asked about their usual consumption frequency of the listed foods, reported as (1) never consumed, (2) number of times per day, (3) number of times per week, and (4) number of times per month. Food photograph maps and molds (expressed at 90 kcal of food weight) were used to assist the participants in recalling their daily food consumption and estimating food weight.

Frequency values were divided by the time interval (weekly or monthly) to convert them into daily consumption frequencies. Nutrient intake was then calculated by multiplying the food weight by the nutrient content, obtained from the Chinese Food Composition Tables (18). For food items not listed in the Chinese Food Composition Tables, data from the United States Department of Agriculture (USDA) was referenced (20). In particular, we did not calculate the nutrient intake of sour soup, while several studies in China have analyzed such soups; most have focused on the diversity of microbial communities (21, 22).

Concerning 24HR, the recipes for the dishes recorded in the recalled diet were analyzed, and the consumption of each dish was disaggregated into its components. The same method as the FFQ was used to obtain the nutrient intake.

### 2.4 Statistical analyses

Statistical analysis was performed using R software (version 4.2.0). The median, first quartile, and third quartile were applied to perform daily intake given the skewed distributions of intake for most food groups and nutrients. The Wilcoxon rank-sum test was used to compare food group and nutrient intake differences between two FFQs, as well as between FFQ and 24HR. In the validity study, Pearson's correlation coefficient (or Spearman's for non-normally distributed data) was used to estimate the correlation size between the two tools and the two FFQs. The magnitude of the correlation coefficient (0 to 1) indicates the strength of the correlation; the correlation coefficient above 0.50 is ideal, between 0.20 and 0.49 is acceptable, and <0.20 is considered poor (23). Adjusted nutrient intake was computed using the residual method in the linear regression model, where total energy intake was the independent variable and nutrients were the dependent variable. All nutrients were subjected to a normal distribution by log transformation before entering the regression model. Considering the influence of random within-person variation in reducing correlation coefficients, the following equation was used to obtain corrected correlation coefficients (24):


$$\begin{array}{l} r_{\text{c}}=r_{\text{o}}\left(1+\lambda x/n_{\text{x}}\right) \end{array}$$

The quartile cross-classification method and Bland–Altman plots were used to measure the agreement between the FFQ and 24HR. Weighted kappa (kw) statistics were calculated, considering the different levels of agreement between categories from the cross-classification table. Values of weighted kappa over 0.60 indicate good agreement, between 0.20 and 0.60 are acceptable, and values below 0.20 indicate poor agreement (23). For the reproducibility study, except for the correlation coefficients, the intraclass correlation coefficient (ICC) was used to measure test–retest reproducibility. ICC values were interpreted as follows: <0.50, poor outcome; 0.50–0.74, acceptable; 0.75–0.90, good; and >0.90, excellent (25). Statistical significance was set at a *p* < 0.05 in all the analyses.

## 3 Results

### 3.1 General characteristics

Initially, 344 pregnant women were recruited, with 153 participants chosen for the validity study and 125 participants for the reproducibility study (dropout rates: 55.52% and 63.66%, respectively). The mean age of the participants in the validity study was 28.91 ±5.46 years and that of the participants in the reproducibility study was 28.74 ± 3.37 years. Exactly two-thirds of pregnant women in the second trimester participated in the reproducibility and validity studies (Table 1).

**Table 1:** General characteristics of participants in reproducibility and validity studies.^a^

		**Reproducibility study**	**Validity study**
*n*		125	153
Age		28.74 ± 3.37	28.91 ± 5.46
Height		154.08 ± 4.11	154.71 ± 5.41
Pregnancy (%)^b^	First trimester	7 (5.60)	11 (7.20)
	Second trimester	79 (63.20)	110 (71.90)
	Third trimester	39 (31.20)	32 (20.92)
Pre-pregnancy weight		52.73 ± 4.87	53.73 ± 8.62
Pre-pregnancy BMI		22.21 ± 1.80	22.51 ± 3.50
Pre-pregnancy BMI categories (%)^c^	Underweight	6 (4.80)	12 (7.84)
	Normal weight	103 (82.40)	91 (59.47)
	Overweight	16 (12.80)	41 (26.80)
	Obseity	0 (0)	9 (5.88)
Family size (%)^d^	Single family	2 (1.60)	2 (1.31)
	Small family	71 (56.80)	87 (56.90)
	Medium family	32 (25.60)	31 (20.26)
	Large family	20 (16.00)	33 (21.57)
Marital status (married, %)		80 (84.00)	133 (86.90)
High education level (%)^e^		49 (39.20)	71 (46.40)
Average monthly household income (%)	<1000	16 (12.80)	13 (8.50)
	1000–3000	62 (49.60)	61 (39.87)
	>=3000	47 (37.60)	79 (51.63)

^*a*^The Kolmogorov–Smirnov test is employed to assess the data's distribution characteristics. For continuous variables that follow a normal distribution, mean and standard deviation are typically presented; For categorical variables, the number and corresponding percentages [n (%)] are displayed. ^*b*^First trimester: less than 13 weeks; second trimester: 14–27 weeks; third trimester: 28 weeks or more. ^*c*^Calculated according to the guidelines for prevention and control of overweight and obesity in Chinese adults (26). ^*d*^Family size the number of individuals living together in a single household, was divided into the following four categories: single family: one person; small family: two to four people; medium family: five to six people; large family: seven or more people. ^*e*^School Year ≥12.

### 3.2 Reproducibility

The median daily consumption of food groups and nutrients from the two FFQs is shown in Table 2. Differences were observed in the soybeans and nuts groups, where daily consumption in FFQ1 was lower than in FFQ2 (*P* < 0.05). The estimated intake of phosphorus, potassium, magnesium, and zinc nutrients in FFQ1 was higher than in FFQ2 (*P* < 0.05). Correlation coefficients ranged from 0.41 (fruit) to 0.71 (vegetables) among food groups, and for nutrients, these coefficients varied from 0.46 (energy) to 0.64 (calcium). The average ICC values were 0.61 in food groups and 0.65 in nutrients.

**Table 2:** Reproducibility of food groups and nutrients between FFQ1 and FFQ2.^a^

	**FFQ1**	**FFQ2**	***P*-value^b^**	**ICC**	**Coefficients^c^**
**Food groups**					
Grains/Tubers (g/d)	395.41 (298.21, 520.18)	428.50 (310.50, 545.20)	0.28	0.65	0.65
Vegetables (g/d)	317.14 (165.71, 529.38)	321.21 (186.76, 540.95)	0.71	0.75	0.71
Fruits (g/d)	288.60 (151.10, 517.40)	325.00 (175.00, 534.14)	0.43	0.37	0.41
Meat/Poultry/Fish/Egg Eggs/Dairy (g/d)	253.00 (135.90, 417.50)	231.60 (142.80, 446.70)	0.27	0.67	0.53
Soybeans/Nuts (g/d)	42.86 (15.20, 123.45)	29.14 (6.83, 97.82)	0.02	0.47	0.50
Sour Soup (g/d)	14.29 (0.96, 57.14)	19.14 (2.19, 57.14)	0.50	0.77	0.53
**Nutrients**					
Energy (kcal/d)	2031.60 (1713.10, 2469.70)	2029.60 (1614.80, 2493.40)	0.68	0.68	0.45
Protein (g/d)	61.85 (47.64, 81.24)	62.33 (46.25, 77.95)	0.54	0.77	0.51
Fat (g/d)	85.58 (72.80, 101.23)	85.55 (69.24, 102.50)	0.66	0.81	0.54
Carbonhydrates (g/d)	251.17 (190.24, 329.03)	253.38 (193.59, 322.73)	0.73	0.54	0.48
Fiber (g/day)	12.19 (8.60, 16.96)	12.55 (8.65, 18.25)	0.51	0.50	0.49
Retinol (ug/d)	182.19 (90.74, 362.50)	212.92 (76.46, 319.16)	0.89	0.49	0.46
Thiamine (mg/day)	0.91 (0.72, 1.14)	0.92 (0.68, 1.24)	0.64	0.73	0.58
Riboflavin (mg/d)	1.04 (0.79, 1.37)	1.04 (0.74, 1.47)	0.76	0.68	0.57
Niacin (mg/d)	17.35 (13.70, 20.92)	17.38 (13.39, 22.94)	0.62	0.71	0.50
Vitamin C (mg/d)	150.70 (91.49, 236.30)	161.03 (101.89, 242.53)	0.60	0.48	0.48
Vitamin E (mg/d)	21.05 (13.22, 37.00)	18.80 (11.45, 27.47)	0.97	0.36	0.36
Folic acid (ug/d)	267.54 (198.24, 369.88)	250.07 (177.86, 366.38)	0.15	0.61	0.61
Calcium (mg/d)	528.93 (375.84, 690.91)	480.39 (337.75, 732.03)	0.51	0.69	0.64
Phosphorus (mg/d)	1009.22 (781.07, 1270.50)	1009.07 (782.02, 1250.04)	0.02	0.75	0.54
Potassium (mg/d)	2482.74 (1882.05, 3291.36)	2366.70 (1840.90, 3447.11)	0.02	0.66	0.58
Magnesium (mg/d)	321.26 (232.75, 399.94)	313.67 (240.56, 433.85)	0.03	0.68	0.57
Iron (mg/d)	17.87 (13.26, 23.11)	17.38 (13.28, 24.07)	0.09	0.64	0.50
Zink (mg/d)	9.49 (7.72, 11.76)	9.82 (7.64, 12.22)	0.01	0.77	0.52
Selenium (mg/d)	32.95 (24.97, 44.11)	32.37 (22.71, 42.47)	0.53	0.72	0.57

^*a*^Data are presented as median, upper, and lower quartiles. ^*b*^Wilcoxon Signed-Rank Test was used to compare the daily intake of food groups and nutrients calculated based on FFQ1 and FFQ2; P < 0.05 was considered as a significance level. ^*c*^Pearson or Spearman correlation coefficients were calculated for normally and non-normally distribution characteristics

### 3.3 Validity

Table 3 displays the median daily intake as assessed by FFQ and 24HR. Except for grains and tubers, sour soup, retinol, thiamine, vitamin C, vitamin E, and selenium, the estimated intake of remaining food groups and nutrients measured by the FFQ was higher than that of the 24HR (*P* < 0.05). Correlation coefficients ranged from 0.11 (vegetables) to 0.54 (meat/poultry/fish/eggs/dairy) for food groups and from 0.19 (vitamin E) to 0.51 (energy) for nutrients. After adjusting for energy and accounting for attenuation, correlations varied from 0.10 (vegetables) to 0.63 (meat/poultry/fish/eggs/dairy) and from 0.15 (iron) to 0.52 (riboflavin).

**Table 3:** Validity of food groups and nutrients between FFQs and 24HR.^a^

	**FFQ**	**24HR**	***P*-value^b^**	**Correlation coefficients** ^**c**^		
				**Crude**	**Energy-adjusted** ^d^	**Energy-adjusted and de-attenuated** ^e^
**Food groups**						
Grains/Tubers (g/d)	380.57 (300.00, 499.03)	350.00 (270.00, 484.20)	0.11	0.43	0.39	0.45
Vegetables (g/d)	342.81 (214.29, 502.05)	250.00 (150.00, 400.00)	<0.001	0.11	0.08	0.10
Fruits (g/d)	350.00 (192.86, 500.00)	213.00 (100.00, 360.00)	<0.001	0.28	0.20	0.23
Meat/Poultry/Fish/Eggs/Dairy (g/d)	256.37 (144.35, 420.88)	205.00 (105.00, 350.00)	0.02	0.54	0.55	0.63
Soybeans/Nuts (g/d)	30.14 (9.56, 83.33)	0.00 (0.00, 37.50)	0.02	0.24	0.22	0.25
Sour Soup (g/d)	21.43 (6.58, 57.14)	0.00 (0.00, 90.00)	0.42	0.45	0.38	0.43
**Nutrients**						
Energy (kcal/d)	2014.64 (2353.41, 3420.92)	1808.00 (1475.00, 2112.00)	<0.01	0.51	-	-
Protein (g/d)	62.73 (48.17, 75)	57.00 (42.40, 72.60)	0.05	0.40	0.37	0.43
Fat (g/d)	86.41 (73.02, 100.98)	86.41 (73.02, 100.98)	<0.01	0.31	0.15	0.20
Carbonhydrates (g/d)	251.17 (196.76, 309.48)	276.6 (212.8, 344.1)	0.02	0.45	0.28	0.32
Fiber (g/day)	12.55 (9.71, 17.51)	7.70 (5.20, 11.00)	<0.001	0.28	0.20	0.23
Retinol (ug/d)	197.79 (99.87, 301.15)	220.00 (92.00, 359.00)	0.14	0.43	0.42	0.48
Thiamine (mg/day)	0.94 (0.71, 1.16)	0.86 (0.61, 1.12)	0.11	0.30	0.18	0.21
Riboflavin (mg/d)	1.07 (0.78, 1.39)	0.58 (0.46, 0.88)	<0.001	0.40	0.39	0.52
Niacin (mg/d)	16.98 (14.09, 21.28)	12.64 (9.88, 15.78)	<0.001	0.31	0.19	0.22
Vitamin C (mg/d)	168.03 (117.79, 235.14)	70.65 (34.75, 123.28)	0.31	0.31	0.35	0.40
Vitamin E (mg/d)	19.06 (12.29, 28.80)	20.25 (16.01, 24.55)	0.24	0.19	0.16	0.19
Folate (ug/d)	269.39 (196.16, 353.36)	123.00 (83.30, 161.50)	<0.001	0.29	0.15	0.17
Calcium (mg/d)	529.42 (372.18, 714.45)	275.00 (161.00, 442.00)	<0.001	0.42	0.41	0.47
Phosphorus (mg/d)	1012.96 (813.68, 1201.14)	751.00 (566.00, 954.00)	<0.001	0.41	0.35	0.40
Potassium (mg/d)	2433.97 (1942.09, 3237.62)	1455.00 (1128.00, 1982.00)	<0.001	0.37	0.28	0.32
Magnesium (mg/d)	318.76 (246.47, 405.61)	224.00 (164.00, 277.00)	<0.001	0.36	0.24	0.28
Iron (mg/d)	17.60 (13.75, 22.28)	11.50 (9.10, 14.40)	<0.001	0.29	0.13	0.15
Zink (mg/d)	9.67 (7.82, 11.84)	8.09 (6.39, 10.51)	<0.001	0.39	0.22	0.25
Selenium (mg/d)	33.10 (24.41, 41.09)	28.27 (20.20, 37.96)	0.36	0.38	0.31	0.36

^*a*^Data are presented as median, upper, and lower quartiles. ^*b*^Wilcoxon Signed-Rank Test was used to compare the daily intake of food groups and nutrients calculated based on FFQ and 24HR; P < 0.05 was considered as a significance level. ^*c*^Pearson or Spearman correlation coefficients were calculated for normally and non-normally distribution characteristics. ^*d*^Data were log-transformed before entering the regression model and the residual method was used to adjust energy's impact on food and nutrient intake. ^*e*^Variance analysis was used to eliminate random within-person variation.

**Figure 3:**
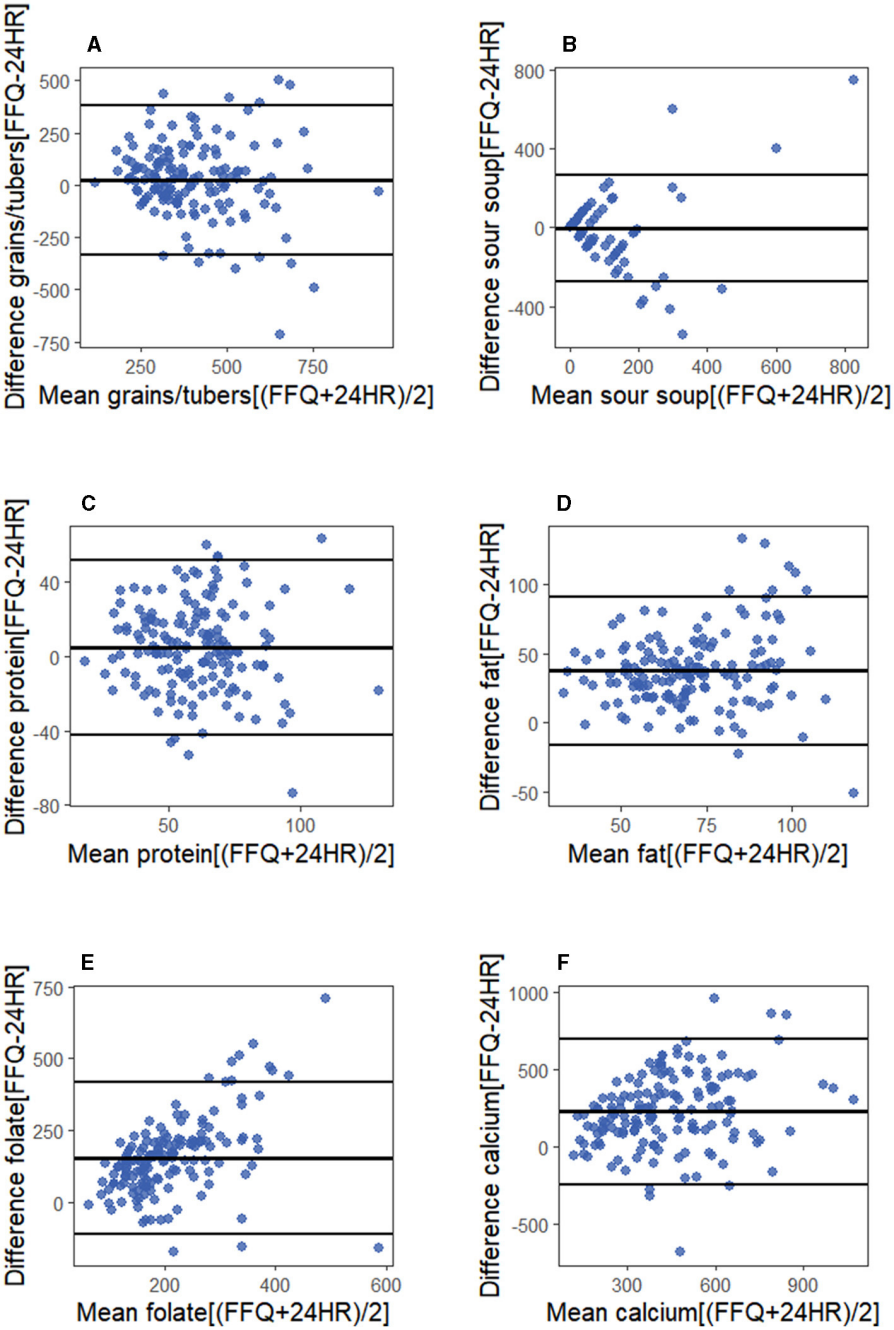
The Bland–Altman plot illustrates the differences through the Food Frequency Questionnaire (FFQ) and two-day 24-hour recalls (24HR) in estimation intake of **(A)** grains/tubers; **(B)** sour soup; **(C)** protein; **(D)** fat**; (E)** folate; **(F)** calcium. The x-axis displays the mean values derived from both methods, y-axis shows their differences. The central line in the plot represents the mean difference between the two methods, with the top and bottom lines indicating the 95% limits of agreement.

Table 4 shows the results of the cross-classification and weighted kappa analyses from FFQ and 24HR. The percentage of pregnant women correctly classified into the same or adjacent quartiles varied from 64.08% (vegetables) to 95.29% (sour soup) among the food groups and from 68.88% (vitamin E) to 78.81% (energy) among the nutrients. Comparatively, 2.01% (sour soup) to 10.56% (vegetables) and 2.65% (energy) to 8.61% (protein, retinol, and folic acid) were misclassified into extreme quartiles. The kw values ranged from 0.03 (vegetables) to 0.30 (grains/tubers) and from 0.10 (vitamin E) to 0.28 (magnesium). Among these, four food groups and 13 nutrients had a kw between 0.21 and 0.60, indicating most of the food groups and nutrients exhibited an acceptable agreement. The Bland–Altman plots for protein, fat, calcium, and folate are presented in Figure 3, as these nutrients are crucial during pregnancy. Additionally, plots for sour soup and grains or tubers, which are featured foods in our FFQ, are included. The results demonstrated that most points fell within the limits of agreement and were clustered around the line of mean difference.

**Table 4:** Cross-classification and weighted kappa analyses from FFQ and 24HR.^a^

	**Percentage agreement of cross-classification (%)**			**Weighted Kappa coefficient^a^**
	**Same/adjacent quartile**	**One quartile apart**	**Opposite quartile**	
**Food groups**					
Grains and Tubers	79.86	15.28	4.86	0.30^**^
Vegetables	64.08	25.35	10.56	0.03
Fruits	74.20	20.97	4.84	0.18^**^
Meat/Poultry/Fish/Eggs/Dairy	81.95	15.28	2.78	0.39^**^
Soybeans and Nuts	87.93	8.05	4.02	0.18^**^
Sour Soup	95.29	2.68	2.01	0.23^**^
**Nutrients**					
Energy	78.81	18.54	2.65	0.27^**^
Protein	76.82	14.57	8.61	0.26^**^
Fat	72.86	20.53	6.62	0.21^**^
Carbonhydrates	74.17	22.52	3.31	0.24^**^
Fiber	74.84	17.22	7.95	0.19^**^
Retinol	72.19	19.21	8.61	0.16^**^
Thiamine	73.52	19.87	6.62	0.19^**^
Riboflavin	73.51	21.19	5.30	0.23^**^
Niacin	72.84	21.85	5.30	0.21^**^
Vitamin C	76.16	16.56	7.28	0.24^**^
Vitamin E	68.88	23.84	7.28	0.10
Folic acid	75.50	15.89	8.61	0.22^**^
Calcium	78.81	16.56	4.64	0.28^**^
Phosphorus	71.52	21.19	7.28	0.17^**^
Potassium	77.48	15.89	6.62	0.24^**^
Magnesium	78.80	15.89	5.30	0.28^**^
Iron	76.16	17.88	5.96	0.25^**^
Zink	75.49	19.21	5.29	0.22^**^
Selenium	75.50	18.54	5.96	0.21^**^

^*a*^Statistics significance level: ^*^P < 0.05, ^**^P < 0.01

## 4 Discussion

Valid questionnaires for evaluating the nutrient intake of pregnant women from ethnic minority groups in China are currently lacking. Hence, we developed a culturally tailored FFQ for pregnant Miao women in southwest China, considering the traditional Miao dietary culture, and assessed its reproducibility and validity in the present study. For reproducibility, our results revealed that the mean correlation coefficients of food groups and nutrients between FFQ1 and FFQ2 were 0.56 and 0.52, respectively, These values were superior to the reproducibility results for pregnant women in Wuhan, China, as reported by Zhang et al. (10) (mean reproducibility for food groups: 0.32, for nutrients: 0.40) and to the reliability of nutrient intake for pregnant women in Shaanxi, China, as reported by Cheng with a reliability of 0.46 (10, 13). However, the ICC was lower than that found in a study of pregnant Lebanese women (27), where the average ICC was 0.96. Notably, the Lebanese study conducted two FFQs within a shorter interval, possibly contributing to the higher reproducibility. A review reported that the interval between FFQ administrations is related to reproducibility performance (28). Short intervals may result in an overestimation of FFQ reproducibility due to potential influence from initial responses. Conversely, long intervals may reduce reproducibility due to changes in dietary habits (28, 29). The 4- to 6-week interval between the first and second FFQ in this study was deemed appropriate (28).

For validity measurements, the average correlation coefficient for food groups in this study was 0.40, except for the vegetable group. The result was similar to or lower than those in validation studies involving Norwegian and Brazilian pregnant women (30, 31) but superior to the results from studies with pregnant women in Shaanxi and Guangzhou, China, which reported average validity values of 0.31 and 0.34 (11, 14). It is noteworthy that a correlation coefficient of 0.10 was observed within the vegetable group, suggesting that our FFQ is ineffective in estimating vegetable consumption. This finding aligns with a prior study indicating the limited ability of FFQ to estimate vegetable intake (32). However, the results are still lower than those of studies in China (10, 14). We suggest that this phenomenon is not solely attributable to the inherent diversity of vegetables, the complexity of Chinese recipes, and the prevalence of communal meals among family members. It is also because our study included pregnant women at different trimesters of pregnancy (7.20%, 71.90%, and 20.92% for the first, second, and third trimesters, respectively). Savard et al. (26) demonstrated differences in vegetable intake among the trimesters, further reducing the correlation between the assessment tools. Another reason is related to the vegetable items in our FFQ. A further investigation by our researchers revealed significant individual differences in the types and frequency of vegetables consumed by pregnant Miao women, especially wild vegetables. Considering the potential impact of questionnaire length on its validity, the FFQ did not include rare types of vegetables although a few participants consumed them; this omission may widen the intake disparities between the FFQ and 24HR.

The average correlation coefficient for nutrients is 0.36, similar to or higher than the values reported in previous studies on pregnant women (14, 33) but lower than those reported by Zhang et al. (10) and Cheng et al. (13). However, their studies did not specify the ethnicity of the participants. A validation study of the FFQ among multi-ethnic adults in northwest China indicated an average validity coefficient of nutrients of 0.52 (34), but its results are not directly comparable to our study. Compared to the general population, the symptoms of nausea or vomiting during pregnancy lead to greater appetite fluctuations (35). These fluctuations may impact pregnant women's responses and the assessment of long-term dietary intake during pregnancy, resulting in weaker correlations between instruments in pregnant women. Our results showed that misclassification rates for both foods and nutrients were below 10% except for the vegetable group, indicating a good interquartile agreement (36). The findings of Bland–Altman indicated that the FFQ tends to overestimate the consumption of important food groups and nutrients. As demonstrated in previous studies on pregnant women, women tend to overestimate their food intake using the FFQ (37, 38). Additionally, FFQ estimates for most of the intake also exceeded those of the 24HR in the validity measurement. This overestimation may be attributed to participants overestimating food consumption frequency or portion intake in the FFQ while underreporting their intake in the 24HR. However, it is important to note that FFQ is intended to rank individuals based on the intake levels of specific food groups or nutrients rather than providing absolute intake values (39).

This study has several limitations. First, our participants were from a county in Guizhou Province, China. Economic and educational disparities compared to urban areas affected participant compliance and cooperation, resulting in a higher dropout rate. For example, some pregnant women may skip routine prenatal check-ups, making it difficult for investigators to obtain more repetitive 24HR. Second, we were compelled to use 2 days of 24HR as reference methods due to cooperation and the limited literacy level of study subjects. Using these limited recalls to assess long-term dietary intake among pregnant women is another limitation, as it may not fully represent the fluctuating dietary habits during pregnancy. This fluctuation could result in disparities between assessment tools; however, these are differences between instruments rather than flaws in the FFQ. Despite these limitations, the present study has strengths. First, pregnant women in the MCCMC cohort from a typical Miao community are known to follow a customary dietary culture. This dietary culture offers a comprehensive and authentic sample that mirrors the dietary preferences of pregnant Miao women. Second, the development of the FFQ food list underwent careful screening, and data were collected by trained surveyors. Face-to-face interviews were conducted for both the FFQ and 24HR, using appropriate visual aids to assist the respondents in recalling the portion sizes of the listed foods.

To our knowledge, the present study represents the first attempt to validate a customized FFQ designed specifically for pregnant women from ethnic minority groups in China. The results indicate that the FFQ demonstrates ideal reproducibility and acceptable validity in estimating and ranking the intake of most food groups and nutrients among Miao pregnant women in China. We are of the view that this instrument will be useful in investigating dietary factors related to pregnancy outcomes among pregnant Miao women in China.

## Data availability statement

The original contributions presented in the study are included in the article/supplementary material, further inquiries can be directed to the corresponding author.

## Ethics statement

The studies involving humans were approved by the Medical Science Ethics Committee of the Affiliated Hospital of Guizhou Medical University. The studies were conducted in accordance with the local legislation and institutional requirements. The participants provided their written informed consent to participate in this study.

## Author contributions

XN: Software, Writing – original draft. TQ: Methodology, Writing – review & editing. RW: Data curation, Writing – review & editing. FW: Investigation, Writing – review & editing. YL: Funding acquisition, Methodology, Writing – review & editing. SW: Writing – review & editing.
